# Ten simple rules for making the supplement increase your paper’s impact

**DOI:** 10.1371/journal.pcbi.1014419

**Published:** 2026-06-22

**Authors:** Volker Grimm, Uta Berger, Stefano Mammola

**Affiliations:** 1 Department of Ecological Modelling, Helmholtz Centre for Environmental Research – UFZ, Leipzig, Germany; 2 University of Potsdam, Plant Ecology and Nature Conservation, Potsdam, Germany; 3 TU Dresden, Faculty of Environmental Sciences, Institute of Forest Growth and Computer Sciences, Dresden, Germany; 4 Molecular Ecology Group (MEG), National Research Council of Italy (CNR), Water Research Institute (IRSA), Verbania Pallanza, Italy; 5 Laboratory for Integrative Biodiversity Research (LIBRe), Finnish Museum of Natural History (LUOMUS), University of Helsinki, Helsinki, Finland; Carnegie Mellon University, UNITED STATES OF AMERICA

## Abstract

Have you ever lost hours navigating supplementary materials—clicking between the main text and dozens of auxiliary files only to encounter broken links, illegible figures, and undefined variables and acronyms? If so, you’re not alone. What should support scientific communication has instead become an obstacle: supplementary information (SI) increasingly suffers from inconsistent formatting, poor accessibility, and fragmented organization that impedes rather than advances understanding. This is disheartening since the SI, if used effectively, has the power to enhance transparency, credibility, and reproducibility of research. Therefore, we propose 10 simple rules to help authors design SI that genuinely increase the impact of their research. The rules emphasize treating SI with the same care as the main text, using it strategically to support the scientific narrative while preserving clarity and focus. Key recommendations include creating a single, well-structured, self-contained SI master document; ensuring explicit cross-referencing between the main text and SI; making SI machine-readable; and avoiding the misuse of SI as a substitute for proper data repositories. We also highlight the importance of creativity in choosing appropriate formats and strict adherence to journal-specific guidelines. Finally, when available, we advocate the use of standardized templates to improve consistency, readability, and reuse across studies. By following these rules, authors can substantially increase the scientific impact of their work while at the same time contributing to more sustainable research practices.

## Introduction

In the old days, when papers were only published in print and before the invention of the internet (yes, those times existed), articles occasionally included appendices with one or two extra data tables. The concept of ‘supplementary information’ (SI) (alternatively referred to as ‘supplementary materials,’ ‘supporting information,’ and similar) had not yet become mainstream. Nowadays, nearly all journals—whether published online only or in print—allow authors to include online supplementary information with their articles, and most authors make great use of this option.

They do so for three main reasons: (1) Further information may contribute to a better understanding of the study. On the internet, there are no severe space limitations, so you can add as much additional information as you want, including the data and code used, the results of additional analyses, or background information about the context and methods of the presented research; (2) The amount of data and the methods used to analyze it have exploded. We usually have to use much more of this additional information to explain what we have done than could ever be included in a paper. (3) Journals are increasingly pushing for short papers, often by imposing strong word limits (typically 1,500–3,000 words for the main text). While this is partly driven by the exponential growth in the number of submissions [1, 2], it also adjusts to the changed reading habits [3], and it certainly helps publishing journals to make more profits. In principle, though, making articles concise and punchy is a good thing [4], but they still should provide sufficient evidence and material to ensure reproducibility of the presented research. This is where the SI comes in.

However, as papers are getting shorter, more and more authors are using the SI as a chaotic “grab-bag” for all the information that cannot fit in the main text [5]. This hinders the advancement of science, complicates the peer review process and prevents a proper understanding of the science behind impressive results condensed into 1,500 words. To revert this trend, some online journals even advise against supplements due to the sobering assessment that “Supplementary material is currently where data and methods go to die, never to be viewed again.” [6].

This is unfortunate because the limited space in the main articles helps both authors and readers focus on the main story. This story would easily be lost if we kept information that nowadays would go to the supplement in the main text, and in many cases articles would become prohibitively long. It is therefore becoming critically important to craft supplementary files properly. However, little guidance is available on how to best achieve this. Most journals’ guidelines for preparing articles only cover the main text, saying virtually nothing about supplementary information except for file types and size (with the notable exception of some publishers). They also do not explicitly ask reviewers to read and evaluate the SI.

**Fig 1:**
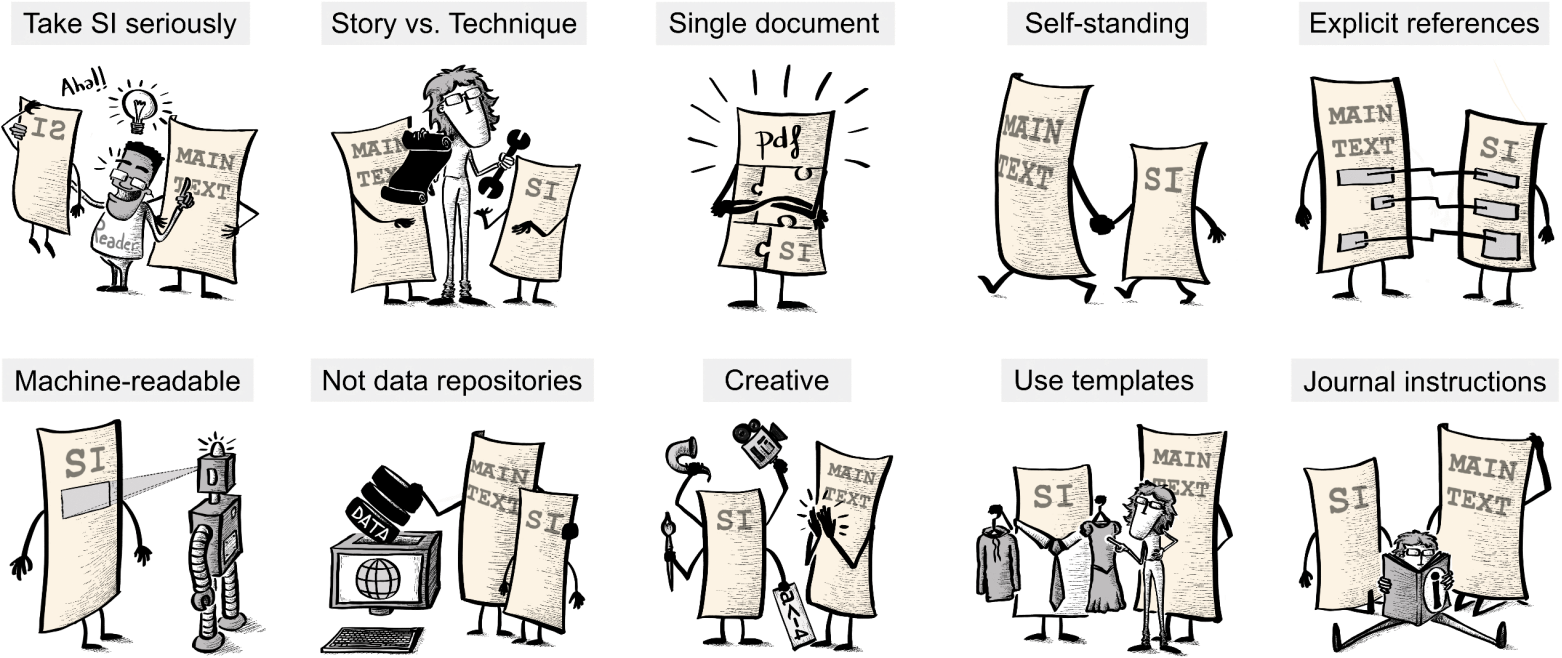
Cartoons illustrating the 10 simple rules for increasing your paper’s impact through the proper use of supplements (see the accompanying text for details). It might be a good idea to print this figure and post it near your workspace (figure by Jagoba Malumbres-Olarte).

The 10 rules that we are suggesting for crafting useful SI (Fig 1) are easy to follow. The effort required is negligible compared to the overall effort involved in conducting the research, but the increase in the credibility, transparency, reproducibility, and impact of your work can be enormous.

Reading the 10 rules, however, might not be enough, since you as an author would still have to make many decisions about the structure of your supplement and where your readers can find a particular type of information. We therefore provide, in Rule 9, a standard template, which, once used in a sufficient number of publications, will make reviewers, editors, and readers expect to see this structure. Such documentation standards have been highly successful across scientific domains, from the PRISMA workflow for reporting of systematic reviews and meta-analyses [7, 8], to the ODD protocol for describing agent-based models [9, 10], along with the Darwin Core Standard for publishing biodiversity data [11], just to cherry-pick a few examples.

## Rule 1: Take the SI as seriously as the main text

They are essential for fully understanding your study in detail. They can trigger those famous ‘aha’ or even ‘Eureka’ moments, encouraging readers to apply your methodology and build their own studies on it. While the main text of your publication presents the story of your study (its background, the research question, the methodology, the results, and the conclusions), your supplementary material provides more detailed insights into the workflow, making your methodology transparent. This significantly strengthens confidence in your work, and presumably your scientific reputation. For instance, presenting your simulation model, or data analysis in a reusable format will ensure your work has a longer-term impact. Prominent examples of well-prepared supplements can be found in Kellner and Swihart [12] and Marxsen and colleagues [13].

Pay attention to your supplementary material throughout the entire process of preparing and submitting your article, and beyond that. There are many examples of papers in which the names of variables, simulation scenarios, and so on, used in the main text do not match those used in the appendices. Unfortunately, Kellner and Swihart [12] is also an example of this. This may occur when final changes are made to the manuscript during revision in accordance with reviewers’ comments, but the corresponding changes are overlooked in the supplements. Also, needless to say, but we are saying it nevertheless: pay as much attention to proper and consistent formatting, to spelling and language checks, and to cross-checking of all references as you do with the main text.

Is there anything more frustrating than a model or script that can be downloaded but cannot be executed? Before compiling the final version, carefully check that all files are up to date. When providing model code, document it carefully and specify the software version under which it can be executed. Readers who wish to reuse your work several years after publication will thank you for this. If you use specific input files (e.g., raster or meteorological data files) that are necessary for executing your model or performing data analysis, provide these together with the model code and your scripts (but see Rule 7 for additional best practices). If you cannot provide the necessary input data due to property rights (e.g., because of your collaboration partners), you should at least prepare data files for a demo version. Providing supplementary material that generates error messages is annoying and reduces the impact of your work.

## Rule 2: For compiling the SI, distinguish the story from technical details

Contrary to what one might intuitively expect of something called ‘scientific’, virtually all textbooks on scientific writing agree that essentially it is storytelling [14, 15]. As outlined in Whitesides’ [16] widely-cited framework for scientific writing, research papers must address key questions: What is the problem? Why are you trying to solve it? How are you solving it? What have you done? Who cares?

The answers we provide are the key message of an article. But, for storytelling to be effective, we must ensure that the message is not lost in a sea of auxiliary information in both the Methods and Results sections. Reviewers and readers are impatient, they will stop reading when the story loses momentum and focus. For the Materials and Methods section this means that readers must get both an overview of what we used and did, and all the technical details needed for full transparency and reproducibility – something extremely important as evidence of issues with scientific reproducibility accumulates [17, 18]. Ideally, all this information would fit into the article, but these days are over, especially in disciplines relying on large data sets and complex computational workflows.

The *guiding principle* for compiling supplements thus is: decide on the key message of your article. Then, for the Methods and Results sections, start describing them in the way you would do in a presentation: focus on the essentials, for example, by referring to the common name of a certain statistical analysis, the aims and scope of a certain workflow, or the data sources you were using. The details on how you made sure the conditions for using that analysis were met, the workflows themselves, and how you processed those data are not important for the understanding and acknowledging the key message, but essential for reproducibility and credibility. They belong in the SI.

## Rule 3: Go for one single document, preferably a pdf

Supplements often comprise numerous files, including data files, model code, supplementary analyses, and comprehensive method descriptions. This can be very frustrating. Who wants to open all these files to find the piece of supporting information (SI) they are interested in? Ideally, there should be one master file called ‘Supporting Information’ in PDF format, so that it is machine-readable (see Rule 6 for details). This file should include a table of contents providing an overview of the entire supplement, ideally with hyperlinks to each section. Each section of the master file should be numbered (e.g., S1, S2, etc.) and start with a short executive summary of its contents. If the section is long, it should have its own table of contents for navigation.

Including certain types of information in the master file may not be possible, such as with very long data tables or videos (see also Rule 8). In those cases, the master file should therefore provide hyperlinks to the files or repositories where this information can be found. Readers should be reminded of how to navigate between files using hyperlinks: press the ‘Alt’ button and then the forward or back arrow.

## Rule 4: Keep all parts of the SI self-standing from the main text

Give your supplementary material a title that relates to the main text, but which also makes it clear that it is a separate document. This will help your readers (including reviewers of your work) to know what to expect when downloading the material and where to find additional information. This could include additional figures; a full description of an agent-based model in ODD protocol format [9, 10]; the code for a null model to evaluate the added value of the more complex model presented in the study; the results of reliability tests; and data and the associated scripts for extracting the model parameters.

Ideally, anyone reading your supplementary material should be able to understand it without having to reread the main text, or even without having read it at all. This means that this document should provide the necessary background information in a concise form, and each element should be understandable in isolation. Any abbreviations used must be introduced here when they are first used. Each figure and table should have a comprehensible caption. It is also important to provide brief explanations of what the reader should see and the conclusions that can be drawn from it. Even the legends must be self-explanatory.

## Rule 5: Have explicit references to the SI in your main text

Your supplementary material provides readers with extensive and valuable additional information. You should therefore ensure that readers find everything as easily as possible. Only then will they appreciate the effort you have put into preparing the material. Ensure that references to the supplementary material (e.g., figures with additional information or results of sensitivity analyses) lead directly to the correct subsection in the supplementary material (ideally with a hyperlink). Use these references in all places in the main text where supplementary material is available. Also include such references in the supplementary material in the opposite direction, so that it is easy to find the relevant sections in the main text from there. Greenbaum and colleagues [19] even suggest a parallel chapter or section numbering for this purpose, which formalizes the relationship between the subsections of the main text and the subsections of the supplementary material.

## Rule 6: Make the SI machine-readable

The increasing use of machine learning, large language models, and other artificial intelligence tools means that scientific papers, and especially their supplementary materials, are now routinely read not only by humans but also by machines. These machine readers are used for tasks such as large-scale data mining, automated evidence synthesis, and contextual summarization. Supplementary materials are particularly important in this respect, as they are often where summary tables and negative or null results are reported, despite these results providing crucial information for meta-analyses and evidence integration [20].

Therefore, ensuring that supplementary materials are genuinely machine-readable is essential for the long-term usability and impact of scientific work. Practical steps include making supplementary files self-contained—by explicitly defining acronyms, variables, and conventions rather than relying on the main text (see Rule 4); providing statistical results in editable tables instead of embedded images; and using vectorized formats for figures containing quantitative information, so that underlying data can be reliably extracted and interpreted without ambiguity. Of course, these practices improve not only computational accessibility but also reuse by human readers.

## Rule 7: Refrain from using the SI as data repositories

As open data has become an essential component of scientific publishing, there has been a growing temptation to release datasets supporting a study in the form of supplementary tables published alongside the article. While this approach may be acceptable for small or illustrative datasets, it is generally considered poor practice for primary research data. Research data should be released in accordance with the FAIR principles (findable, accessible, interoperable, and reusable [21]). However, journal appendices and supplementary materials often fail to meet these criteria for human and machine use (see Rule 6).

In most journals, supplementary data do not receive an independent DOI and lack the rich metadata required to support discoverability and long-term access, which undermines the findability and accessibility principles. Moreover, data published as supplements are rarely subject to requirements for standardized formats, controlled vocabularies, or clear semantics, limiting interoperability and reuse. As a result, such data are often difficult to integrate into secondary analyses, meta-studies, or automated workflows.

We therefore recommend prioritizing certified data repositories for data publication (e.g., Dryad, Figshare, OSF, GBIF, GenBank, Zenodo). These repositories are designed to support persistent identifiers, standardized metadata, and community conventions, and can be reliably cited from both the main text and the supplementary materials (ideally with hyperlinks connected).

Source code, Jupyter notebooks, etc. can also be stored separately, provided they are linked to the SI master file via hyperlinks. Storing model code on GitHub, for example, provides an excellent way to obtain a DOI for the model through a link to Zenodo, and is a convenient option for keeping it up to date.

## Rule 8: Be creative with the SI

For centuries, scientific papers were constrained to static formats—printed text, tables, and figures—reflecting the limitations of print-based dissemination. The internet, however, is now the primary medium through which supplementary materials are distributed and offers far greater flexibility in the types of content that can be shared. Supplementary materials are no longer limited to static PDFs (see Rule 3): they may include audio recordings, videos, executable scripts, interactive visualizations, or three-dimensional models that can be explored dynamically.

Such formats should not be used for novelty alone; rather, they should be used when conveying information that cannot be effectively communicated in static form. For instance, videos can clarify experimental procedures or serve as central evidence in a given piece of research (e.g., a case study on a new animal behavior). Annotated code can document analytical workflows and interactive models can make complex structures or processes far more accessible. Provided the journal allows it (see Rule 9), authors should consider taking advantage of this expanded range of formats to enhance transparency, understanding, or reuse.

## Rule 9: Make use of available templates

Using templates or standard formats can be an efficient way of facilitating the writing and reading of documents. We are all familiar with the standard IMRaD structure of scientific publications: Introduction, Materials and Methods, Results, Discussion, and Conclusion. Templates have also been established for supplements in certain application domains. Examples include the TRACE document for mechanistic/dynamic modeling [22], the ODMAP protocol for documenting species distribution models [23], and the “stepFD” protocol for functional ecology [24].

So, while writing and finishing your paper, make sure you use any existing templates in your field of study when compiling. If there isn’t one, perhaps you could team up with colleagues and create one? Alternatively, you could use the template we suggest in the SI of this article in the meantime. This template implements several rules for creating a master document that can be converted to PDF. It helps to make your SI a standalone document and suggests a table of contents. It also explains how hypertexts are used and includes hyperlinks to further documents. When using this template, please refer to this article as the source. This is important so that we can monitor its use and update it accordingly if needed. This monitoring was critical to the success of the ODD protocol, which was updated twice [9, 10, 25].

## Rule 10: Follow the journal’s instruction

Publishers vary widely in the degree of freedom they allow for supplementary materials, particularly with respect to structure, length, formatting, and citation practices. Some journals impose very few or no constraints, whereas others adopt highly detailed and restrictive guidelines. For instance, several journals published by Cell enforce specific requirements regarding formatting, the number and type of supplementary items (e.g., figures and tables), and even the way references must be cited.

Other journals allow greater flexibility but still impose targeted constraints. For example, journals specializing in reviews and synthesis papers, such as Biological Reviews, often require that all references cited in the supplementary materials also appear in the main bibliography. This policy is intended to ensure that the literature being synthesized receives appropriate visibility and credit [26–28]. Finally, there are even journals that discourage the use of supplements entirely, urging authors to use this option exclusively for very specific types of outputs (e.g., videos; see Rule 8) [6]. It follows that authors should always carefully adhere to journal-specific guidelines for supplementary materials, as such requirements take precedence over any general best-practice recommendations – including the 10 rules discussed here.

## Discussion

The scientific method is defined by full transparency about the methods and materials used, enabling others to replicate the described work if necessary, and by clearly separating the presentation of results from their interpretation. Although this request for transparency and reproducibility appears absolute and rigorous, science and how it is reported are carried out by people and are therefore subject to cultural and normative influences.

The digital revolution has made providing SIs the norm in writing, publishing, and reading publications. The current culture is that, except where templates for SIs exist, nobody knows what to expect when checking an SI. This culture is clearly being accepted—everybody does it—but we can do better.

To drive a cultural transition, one cannot rely on altruism. Scientists face intense pressure in a highly competitive field where building a career is challenging—so who has the time and energy for producing high-quality SI figures or meticulously formatting extensive supplementary tables? A cultural transition, we believe, will happen only if there are direct, concrete benefits for individual scientists. When formulating the 10 rules presented here, we kept this idea in mind. The proposed rules are very simple—some may even come across as elementary to more experienced writers—but the benefits in terms of credibility, transparency, reproducibility, and therefore the potential impact of your work, will be enormous. An additional indirect benefit of following these rules will be that, as more and more people follow them, we will find more and more SI that are well organized and designed, allowing us to better understand and use the work presented for our own future projects.
